# Science under siege: protecting scientific progress in turbulent times

**DOI:** 10.1242/dmm.052348

**Published:** 2025-03-13

**Authors:** James Briscoe, Craig E. Franklin, Daniel A. Gorelick, E. Elizabeth Patton, Michael Way

**Affiliations:** ^1^ Editor-in-Chief, Development; ^2^ Editor-in-Chief, Journal of Experimental Biology; ^3^ Editor-in-Chief, Biology Open; ^4^ Editor-in-Chief, Disease Models & Mechanisms; ^5^ Editor-in-Chief, Journal of Cell Science

As Editors-in-Chief of The Company of Biologists' journals, we have been watching with growing concern the policy changes in the United States of America (USA) and the challenges that these are creating. It is unusual for us to comment on a nation's science policies or politics, but the rapid ongoing developments in the USA and their potential impact on research worldwide are beyond the usual.

Historically, the USA has been the world's leading supporter of scientific research, with the US government being the major funder. This investment has been transformative: from the atom to the genome to the internet to life-saving therapeutics – the USA has led the world. Last year, the National Institutes of Health (NIH) and National Science Foundation (NSF) budgets totalled more than $55 billion, of which around $50 billion went to the medical and life sciences (see https://www.hhs.gov/sites/default/files/fy-2024-budget-in-brief.pdf and https://www.nsf.gov/about/budget/fy2024/appropriations).

The scale of disruption to science funding in the USA is unprecedented. At the NIH, study sections, funding councils and project meetings have been cancelled. Without these, new grants cannot be funded or renewed. Fellowship programs have been cancelled. The jobs of thousands of employees across the government science agencies have been terminated, creating operational paralysis and confusion over and above personal costs. The proposed cuts to indirect cost payments to universities and institutions threaten to create insurmountable funding gaps. This is all part of an explicit effort to radically reduce the size of the US government and to downsize the workforce.

Not only is it the direct effect of these cuts, it is also the uncertainty created by these rapid policy changes that is damaging to the scientific community. Researchers, clinical trials and public health agencies need stable, predictable funding environments to plan and execute long-term studies. The current atmosphere of confusion and unpredictability makes it difficult for scientists to make informed decisions about their research programs and careers. Even if policies are reversed or blocked in the coming months, the damage already done will take years to repair. The loss of experienced personnel, disruption of ongoing research and erosion of institutional knowledge cannot be quickly restored. This cascading effect means that even a temporary period of instability can have repercussions that extend well beyond the immediate crisis.

The dismantling of programs aimed at ensuring equal opportunities for people with disabilities and from underrepresented backgrounds is troubling. This comes at a crucial moment when we are finally seeing meaningful progress in broadening participation in science. The cancelled programs have expanded the talent pool for scientific research and enriched the scientific enterprise by fostering innovation through diverse perspectives. Rolling them back threatens to reverse hard-won gains, and it risks squandering the potential contributions of historically underrepresented groups to scientific discovery.

It is particularly disappointing to see some non-governmental organisations appearing to adopt similar policies, even when not required to do so by federal mandate. While the situation is complex even for those not reliant on federal support, these institutions have historically provided stability during periods of political upheaval, and their independent leadership is needed now more than ever.

The impact on public health is a major concern. Defunding ongoing research initiatives threatens to stall years of progress, both in the USA and internationally. Programs that have saved countless lives through vaccination initiatives and through HIV/AIDS prevention and treatment are being cancelled. Scaling back these initiatives and cancelling research that will underpin future programs is not only ethically questionable, but also economically short-sighted. The long-term costs of decreased public health interventions – both in terms of human lives and economic burden – far outweigh any short-term financial savings.

At the NSF, recent developments have particular significance for climate science research. The timing could not be worse. The world faces unprecedented environmental challenges. The NSF's support for climate science, environmental monitoring and sustainable technology development has been fundamental to our understanding of environmental challenges. Interference with this work – whether through direct funding cuts or censorship – threatens to undermine international efforts to address the climate crisis at a time when accelerated action is desperately needed.

But it is not just about the funding cuts. Our journals support open science and promote scientific rigour. We are concerned with policies that appear to threaten censorship, restrictions on terminology and the removal of public datasets. We are committed to maintaining editorial policies, scientific standards and data openness in our journals. We are determined to protect inclusivity, scientific rigour and the peer review process.

US government funding has been instrumental in supporting leading scientists, fostering international collaboration and maintaining crucial research infrastructure – from databases to irreplaceable collections. The potential decline in US global scientific leadership in these areas could have far-reaching consequences for international research relationships and collaboration.

To our colleagues in the USA, we stand with you during this challenging period. The international scientific community recognises your contributions and the difficult circumstances you now face. Science is a global endeavour, and setbacks to research in one nation affect us all.

In these uncertain times, we must strengthen our international scientific networks. We must build new bridges of collaboration. We must speak with a unified voice in support of evidence-based policymaking and scientific freedom. Together, we can create a more resilient scientific community, with policies that strengthen rather than diminish research capacity. Science must transcend national boundaries and short-term political considerations.

